# Factors Influencing Rural Hospitals to Participate and Remain in Accountable Care Organizations

**DOI:** 10.1111/jrh.70179

**Published:** 2026-06-10

**Authors:** Emily J. Lawton, Jenah McCarty, Thomas E. Vaughn, Fred Ullrich, Keith J. Mueller

**Affiliations:** ^1^ Department of Health Management and Policy University of Iowa College of Public Health Iowa City Iowa USA; ^2^ Clinical Excellence Business Operations Division Intermountain Health Salt Lake City Utah USA

**Keywords:** accountable care organizations, hospitals, Medicare Shared Savings Program, population health, quality of care

## Abstract

**Purpose:**

Little is known about factors driving rural providers**—**who face unique challenges**—**to participate and remain in accountable care organization models. The purpose of this study was to investigate the specific factors that rural hospitals evaluate when weighing both initial and continued participation in Medicare as well as commercial ACO models. Furthermore, we explored policy implications to improve ACO models’ capacity to attract, retain, and promote success of rural providers.

**Methods:**

Semistructured, in‐depth interviews were conducted with rural hospital executives with direct knowledge of ACO agreement terms and factors driving ACO participation. The interview guide contained seventeen open‐ended questions. Interviews were recorded, transcribed, coded using codebooks informed by the interview guide, and analyzed using a thematic analysis approach.

**Findings:**

Interviewees identified five primary multi‐faceted motivations for participation: (1) financial incentives—shared savings, upside‐only risk initially, and upfront funding for some providers, (2) getting ahead of the curve of the move to value‐based reimbursement, (3) importance placed on capacity to push toward improving population health or quality, (4) additional forms of resource gain, and (5) key outside factors. The specific ACO design affects rural providers’ decision‐making on timing and selection of particular models. Importantly for future policy development, multiple respondents raised concerns about moving to risk in ACO models, leading them to consider dropping out.

**Conclusions:**

Findings from this qualitative study provide an in‐depth understanding that is vital to achieving CMS's goal of increasing participation in value‐based care. In turn, this could further improve health care quality for countless lives.

## 1 Introduction

One of the most important changes to the health care system over the last two decades has been the shift toward value‐based care—paying providers based on quality performance and cost reductions. The Centers for Medicare & Medicaid Services (CMS) had previously set a goal for all traditional Medicare and the majority of Medicaid beneficiaries to be in accountable care relationships by 2030 [1]. Despite this, the number of ACOs, attributed beneficiaries, and hospitals participating in the Medicare Shared Savings Program (MSSP) has remained relatively flat since 2018 [2]. Moreover, rural providers face unique challenges, such as limited health care infrastructure and smaller patient populations. This contributes to lower ACO participation rates compared to urban counterparts. Consequently, it is vital to understand the factors driving rural providers to participate and remain in ACO models—and in turn, achieve quality and cost improvements. This has important practical implications for rural health—in terms of the design of and responsiveness to accountable care organization policies and practices to improve health care quality and population health.

Various Medicare, Medicaid, commercial ACO initiatives and policy revisions have affected providers’ decision‐making to enter in ACO arrangements. The MSSP began in 2012 and grew to be the largest CMS model [3, 4, 5]. Changes introduced in the CY2023 final rule allow new and inexperienced MSSP ACOs to remain in an upside risk model for the initial 5‐year agreement with an additional, optional, 2 years (totaling 7 years without downside risk). This, and other rule changes, may make ACO participation more attractive to rural entrants [6].

National organizations have also played a significant role in the growth of the ACO model. Organizations like Signify (acquired by CVS in 2023) and Aledade have enabled ACO creation by providing initial and ongoing technical and analytic support. They have also combined previously unaffiliated health care providers and facilities in order to achieve attributed beneficiary scale.

Previous research has revealed provider and geographic characteristics associated with ACO participation and factors affecting performance. For example, hospitals in competitive health care markets, especially in metropolitan areas, are more likely to participate in the MSSP. Additionally, having more health information technology applications is positively associated with MSSP participation [7]. Hospitals with previous risk‐based contract experience are better positioned to manage financial risks associated with ACOs, leading to higher participation [8]. Organizational support, through membership in a health care system, increases hospitals’ likelihood to engage in ACOs. Rural hospitals are less likely to participate, reflecting challenges in meeting ACO requirements and achieving scale necessary for effective participation [7]. However, little is known about the factors influencing rural hospitals that do participate in ACOs.

## 2 Methods

### 2.1 Sampling

The universe of hospitals participating in an ACO was identified using data from Torch Insight [9], a national health care database and analytics platform originally developed by Leavitt Partners. The database was used to identify hospitals with commercial and/or Medicare ACO contracts between 2019 and 2021. Our focus was on individual facility decision‐making regarding ACO participation, so hospitals identified as owned by a larger system were not considered. American Hospital Association annual survey data [10] were used to ensure only non‐Federal government general medical and surgical hospitals were retained in the pool.

A total of 1861 distinct, eligible hospitals participating in 580 unique ACOs were initially identified. Of those, we identified 305 (187 CAHs and 118 PPS hospitals) as rural. Hospitals located in rural areas were identified using Rural‐Urban Commuting Area (RUCA) data [11]. Only hospitals in a ZIP code with a RUCA of four or more were retained in the sample.

The agreement histories of the remaining hospitals were examined, and hospitals were subsequently assigned to one of four classifications for commercial and Medicare ACOs:

1. Currently in the MSSP, not in a commercial ACO
2. Currently in both the MSSP and commercial ACO(s)
3. Currently in commercial ACO(s), not in the MSSP
4. Recent exits from the MSSP, not in commercial ACO(s)

Restricting the universe to hospitals in one of these four categories, a sample of 80 hospitals was randomly drawn with stratification on Census region and ACO participation classification as described above.

Lead personnel (CEO, CFO, and COO) at the hospitals in the sample were initially identified using information from the American Hospital Directory website [12] and were confirmed using information from individual hospital websites. A variety of avenues were used to attempt to contact hospital leadership:

- Email addresses for a majority of the hospital contacts were purchased from a commercial vendor.
- Contact lists were provided to colleagues at the National Rural Health Association (NRHA) who were asked to provide introductions and email addresses to “known” constituents.
- Personal and professional network contacts at other state and regional organizations were employed to provide introductions and email addresses to “known” constituents.
- A minimum of three email contacts were made to establish an interview appointment.
- A minimum of four telephone attempts were made to nonrespondents to try to establish an interview appointment.
- A final email request for participation was sent from the NRHA COO to all nonrespondents.

We were unable to establish contact with the leadership of 56 hospitals and had refusals from another 5 hospitals. Both “refusals” and “no contact” hospitals were relatively uniformly distributed across the four hospital classifications. Interviews were conducted with the leadership from 19 (23.8% of the sample):

1. Currently in MSSP, not in commercial ACO: *n* = 5 (26.3%)
2. Currently in both MSSP and commercial ACO(s): *n* = 2 (10.5%)
3. Currently in commercial ACO(s), not in MSSP: *n* = 6 (31.6%)
4. Recent exits from MSSP, not in commercial ACO(s): *n* = 6 (31.6%)

The respondents were also geographically dispersed across all Census regions.

### 2.2 Interviews

We conducted semistructured, in‐depth interviews with rural hospital executives with direct knowledge of ACO agreement terms to understand specific factors rural hospitals evaluate when considering new or ongoing participation in ACOs. An interview guide (available from the corresponding author) was developed focusing on known areas of concern for hospital ACO participation. It contained open‐ended questions about influencing factors for initial participation, and considerations for remaining in or leaving ACOs. We also sought insights on proposals to improve ACO participation, retention, and performance. Finally, we asked for policy recommendations to further help ACO models attract, retain, and promote the success of rural providers.

### 2.3 Analysis

Interviews with leaders representing the 19 rural hospitals were completed, recorded, and transcribed to ensure accuracy. To promote candor, interviewees were guaranteed confidentiality as to their and their organization's identity. Responses were coded and analyzed using NVivo. We used the six‐step thematic approach outlined by Braun and Clarke [13, 14] to analyze the data from the interviews. The six steps include familiarization with the data through readings of transcribed interviews and noting initial analytic observations, initial coding, searching for themes, reviewing themes, defining and naming themes, and writing the analysis. At the start, all study authors read through the transcripts to familiarize themselves with the data and begin the process of identifying themes. While reading the transcripts, the first four study authors noted analytic observations and patterns and created memos on initial reflections on these patterns. All study authors then met to discuss the patterns and develop a codebook with definitions for each code, where codes capture both a semantic and conceptual reading of the data. A full version of the codebook is available on request. The codebook was entered in NVivo. All four coders then used this codebook to separately code all of the interviews, going through each text segment in the given transcript and assigning the relevant code(s) in NVivo, to capture important features of the data relevant to the broad research question. As the coders identified patterns, we described the nature of each theme as well as the relationship between themes. We analyzed, defined, and named each theme, identifying its essence, and we elucidated the given theme with direct quotes. To ensure rigor throughout the entire process, the investigators independently analyzed the data, met regularly to discuss the process and compare findings at every stage, and reached agreement on the analysis.

All protocol methods and interview instruments were reviewed by the university IRB. The project was deemed “Not human subjects research.”

## 3 Study Results

Interviews revealed most decisions to join an ACO were not explicitly driven by existing strategic plans. In the less common cases where plans did guide the decision, organizations viewed ACO participation as a step toward population health goals and potential financial returns.

### 3.1 Population Health or Quality

Rural hospital leaders frequently cited the ability to improve population health or quality as a key motivation for joining an ACO, a priority for rural providers who see significant benefits in patient‐centered care through ACO participation [15]. Several noted that this focus was especially important when succeeding in an ACO meant *“demand destruction”* or losing revenue for already financially vulnerable institutions. As one respondent explained, *“keeping people well and keeping them out of the hospital is just a better perspective for your entire community.”* Respondents also valued the increased emphasis on measuring and improving quality. One leader shared, *“From a quality perspective, if you're interested in caring for your patient population, and using hard data…to do that, I don't know how you can go wrong with the ACO.”* While the increased availability of data from payers and specific quality measures helped organizations identify strengths and areas for improvement, several interviewees noted challenges with the timeliness of data, which limited their ability to respond most effectively to improve performance.

### 3.2 Additional Forms of Resource Gain

Several respondents emphasized the resources gained through joining an ACO, including the ability to form a coalition of providers, learn from peers, and improve care quality or access for their communities. As one respondent described, “*a significant upside was this unified group of providers across a very huge and underserved area…we were all just trying to keep healthcare access going.”* Others highlighted the practical support ACOs provided, such as assistance with MIPS reporting and the ability to report as a group, which reduced administrative burden, along with the ability to identify high‐performing peers based on benchmarking quality across providers. One respondent noted that this support facilitated seeing “*where we sit from the quality matrix,”* which helped them learn from hospitals performing well. For another respondent, switching to a different ACO was a way to access greater support and more engaged partners: *“We would have had to invent those resources ourselves and we just didn't have the time…I don't feel like we're in it alone.”*

### 3.3 Financial Incentives

Another key motivation for joining an ACO was the potential to earn shared savings without initial downside risk. Several leaders described this feature as instrumental in obtaining board approval. As one respondent noted, *“when this was presented to us, it felt like no harm, no foul.”* Others anticipated that participation could help recover Medicare dollars and strengthen their bottom line. However, many later found that the work required to succeed in an ACO could outweigh the financial benefits, with one leader reflecting, “*I thought this was going to be a new revenue stream…well, no.”*

Upfront funding [16, 17] also played a significant role in enabling rural hospitals to participate in ACOs. One leader shared, *“A lot of money was actually given to us to launch these ACOs and there wasn't any risk when we first started.”* Another noted that the availability of grant funding for health coaches was their primary reason for joining. While one hospital earned over $10,000 in shared savings in its first year, the CFO emphasized that implementing the necessary changes independently would have cost *“in the neighborhood of $100,000.”*

### 3.4 Getting Ahead of the Curve

Perceiving a shift toward value‐based reimbursement in the policy environment, many respondents aimed to get ahead of the curve and gain experience through ACO participation. As one hospital CEO stated, “*Yeah, this is the future. Let's start working on this, learning what we're doing, and get prepared for the future.”* For another leader, this perspective, combined with financial incentives, was central: *“If you think that the world, the government and payers are going to be going in this direction because it's the right thing to do, either you can…be part of the solution and share in some of the gains from those improvements, or you can just let somebody else do it and they get all the benefits and our reimbursement just goes down.”*

### 3.5 Key Outside Factors

Several respondents pointed to key outside forces as another driving factor for joining an ACO. For some, Medicare Access and CHIP Reauthorization Act of 2015 (MACRA) requirements made ACO participation an attractive way to reduce reporting burden [18, 19]. As one respondent shared, *“It also helped us meet a lot of our quality goals…it made it easier to report on our quality.”* Others emphasized the role of MIPS in their decision, noting that joining an ACO helped avoid penalties and simplified compliance. One leader explained that they could either *“have gone down [the MIPS and MACRA] scenario”* or choose an ACO pathway that allowed them to *“learn from one another…in a safe space.”*

### 3.6 Design of Specific Models and Policy Changes Recommended

These factors also shaped respondents’ decisions about when to join an ACO and which model to select (Table 1). Most underscored the importance of model design in shaping their experiences and determining whether to continue participation. Respondents further emphasized that the financial vulnerability of many rural hospitals limits their ability to invest in prevention and population health, even within ACOs. In turn, respondents recommended policy changes to improve ACO effectiveness and better attract and retain rural providers (Tables 2 and 3). Given both the tenuous financial position of rural hospitals and the substantial costs associated with ACO participation (including potential losses, necessary care delivery investments, and reduced revenue), the specific ACO model design and risk level are critical.

**TABLE 1:** Key themes and representative quotations from interviews with rural hospital executives about factors driving decisions on timing and selection of specific ACO models, 2023–2024.

Themes			Quotations
**Initial timing and model selection**	Beginning with the MSSP	Many respondents began with the MSSP because it was an early option and offered initial upside‐only risk	*“We decided that it was critical that we get ahead of the curve.…That's why we joined the MSSP Track 1…because we had no idea about our opportunities or weaknesses…it was really just an opportunity to do a deep dive and figure out…where we wanted to move.…After [understanding] the foundation…we could comfortably move [into commercial ACOs].”*
	Later joining commercial ACOs	Respondents described joining commercial ACOs after gaining familiarity with ACO models and seeing more attractive financial designs	*“[They'd] been in a Medicare ACO, and so they're used to focusing on quality…they felt like ‘We're doing it now in the Medicare space; we might as well…see what we can do on the commercial space.’”*
	Remaining only in the MSSP	Some remained only in the MSSP due to concerns about overextending their organization or because of a large Medicare population	*“Medicare shared savings was…the least intimidating and the…right fit for our population”*
**Challenges for rural providers**		Fragile financial positions: Respondents emphasized that rural hospitals’ financial vulnerability makes it difficult to invest in prevention and population health	*“There's a need to develop appropriate compensation models for prevention, but rural hospitals face the challenge of their fragile financial positions.”*
		Concerns about risks of losses: Many expressed concern that downside risk could jeopardize already‐thin margins	*“You would get a lot more involvement in an ACO [if there were no losses]…for critical access hospitals,…the goals of the ACO [can push against] the lifeblood…[of the] hospital.”*
**Importance of model design for continued participation**	Concern about moving toward risk in the MSSP	Numerous respondents considered dropping out or working with an ACO management organization to absorb losses	One respondent who later began working with an ACO management organization shared: *“There just wasn't enough benefit for us to take any risk.”*
		Exiting ACO models	A respondent who decided to exit ACO models shared: *“[That's] why the board doesn't have any interest in trying to participate with ACOs in the future…We run a very lean profit margin, if any…[keeping] our volumes up…to just break even.”*
	Additional factors that have shaped decisions regarding in which models to continue	Differences in risk between commercial and Medicare models: Respondents noted that Medicare downside risk was often higher than commercial risk	*“None of us have an appetite…[for] downside risk, and the magnitude…in the Medicare shared savings is not insignificant.”*
		PMPM payments: Some commercial models offered per‐member per month (PMPM) payments that supported participation	*“In the early days, the way we looked at it was ‘There is some downside risk…But we could pay for it, most likely out of future PMPM payments.’ We had six different contracts and we believed correctly that the likelihood of all of them ending up in the negative seemed low…But I would say that that everything I'm telling you is gradually falling apart…it would be impossible to describe how dire the [financial] situation is for hospitals in our region right now.”*
		Financial design can affect the level of support across providers for participation	*“Primary care and pediatric staff were engaged because they were getting PMPM dollars. The specialists not so engaged. There didn't seem to be anything for them.”*
		CAH cost‐based reimbursement may be a better fit with some commercial ACO models	*“[Our current commercial ACO contract has] been very favorable…There's some financial reward for the work…which we haven't really seen from the Medicare program.”*

*Source*: Authors’ analysis of qualitative data from semistructured interviews of rural hospital executives.

*Note*: MSSP is Medicare Shared Savings Program.

**TABLE 2:** Key policy recommendations from rural hospital executives to (1) improve ACO effectiveness as well as (2) further attract and retain rural providers in ACO models, 2023–2024.

Categories	Suggestions	
***The most prevalent recommendation, expressed by at least seven respondents explicitly and implied by other respondents as well, was to reduce the risk rural providers face to participate***		
**Risk**	Slow or modify transitions to downside risk	*“The preparation for risk and CMS [taking] a step back from forcing all the ACOs into a risk model…has enabled participation to continue.…If the ‘Pathways to Success’ would have continued on in that arena, there would have been a lot of ACOs that would have dropped out.”*
	Eliminate or substantially reduce downside risk for rural providers	*“Remove the risk…people are more likely to get involved and…move the needle on care.… So more carrot, less stick.”*
	Tailor risk expectations for smaller or cost‐based rural providers	*“Downside risk is just going to drive participation to the ground, and there has to be more upside for the smaller hospitals.…I totally understand having some skin in the game, but not to the point of that could put your organization in jeopardy.…[There] has to be a way to…work around that.”*
		*“If they would come up with a rural model that allowed for no downside financial risk…If they require us to go risk, no CEO with our cost‐based reimbursement is ever going to do it”*
	Address escrow requirements that create cash‐flow barriers and more fully reward hospitals for investing in doing the right thing for their community	*“We would love to be in a higher risk.… But the escrow is a deterrent.…Rural hospitals have cash flow issues.”* (An escrow account requires a sizable amount of upfront capital.)
		*“We're in a rural market, but it's a highly competitive market. We're a rural, not for profit community‐owned hospital, and [relatively nearby] is a privately held for‐profit managed hospital that is…not in an ACO, not trying…to do the right thing for their community. And there's no distinction. We don't get any other consideration for the investment we've made in the ACO. At the end of the day, we're left with all this money tied up in escrow.”*
***The next most prevalent recommendations—each expressed by two to five respondents***		
**Incentives and reimbursement**	Strengthen incentives for prevention and avoiding unnecessary care; ensure hospitals are not financially disadvantaged when other stakeholders benefit	*“The secret sauce is figuring out how to compensate hospitals for not doing things. Pay us like crazy to do one thing, but you won't pay us a dime to prevent it. That's where the challenge lies, and there's reluctance from payers in that regard.”*
		*“[Patients, physicians, and Medicare all benefit, but]…the hospital is the one leg of the chair that's upside down on this deal.”*
	Expand access to upfront funding	*“We started…the year they just ended the AIM funding.…We've put a significant amount of money in…when we weren't getting anything back.”*
	Align incentives to avoid undermining quality	*“These critical access hospitals…have…swing beds, and that's basically the same in nursing home as skilled care…A skilled care day is less than $400, but you come to my hospital for a swing bed day…it's $1,200. We know we provide a better product…it's proven year after year…on patient outcomes. But if you're trying to lower the costs on your ACO beneficiaries, you shouldn't put them in a hospital swing bed.…So, I think some of the incentives are backwards for rural America and critical access hospitals.…I've seen people do some crazy stuff to save money.…Some of those incentives might actually backfire in the long run.”*
	Adjust reimbursement to increase buy‐in from certain types of providers, such as rural health clinics	*“When a provider has to put that much time and effort…to not get reimbursed for it because you only get your all‐inclusive rate…It's hard to sell that.…So there needs to be some consideration on how they reimburse for that if that is going to be the way that you attribute a patient to your provider for your total number. Because that…sinks your battleship right from the start. And then you don't have provider buy in.”*
**Data access**	Improve access to timely, transparent data	*“Sometimes…it's this black box…your performance should be clear…you can't be six months late.…It's got to be transparent as close to real time data as possible so you can make good decisions.”*
		*“Get your data up front before you agree to any type of risk…otherwise, you're just…flying blind.”*
		*“With [a commercial payer]…I would like to have better control over the claims to see that.…It's their database…we don't have as much access.”*
**Quality measures**	Modify quality measures to reflect rural context; Standardize metrics and portals across payers; ensure measures are feasible	(1) *“[Come] up with measures that…work [in] the rural community because it is completely different”* (2) *“Have all the payer programs use the same portal [and] metrics”* (3) *“Some of the criteria…are not even reasonable.…We can try all we want but…we're not going to make it there.”*
**Account for unique rural challenges**	Create rural‐specific ACO model options	*“Most of the rural hospitals—the critical access hospitals with the RHC clinics—didn't fit into their ACO picture. So, they made the separate rural ACO.”*
		[We switched ACOs because] *“the really large ACO was not a good fit for us as a rural [provider].…We were just one of many and they just ignored us.”*
	Provide more resources to address scale issues	“*The biggest challenge for rural places is…scale…the law of small numbers*”
**Support patient access to care**	Eliminate co‐pay for chronic care management (CCM)	*“So many people [could] benefit from CCM, and it is the ones that cannot afford it that are really the most at need.”*
	Increase flexibility in care delivery timing	*“With Medicare, you can't bill for a well visit and an encounter the same day.”*
	Allow more flexibility to use mid‐level providers	*“[Requiring that] a patient has to see a doctor where…there is no doctor available…that is unfair to the system, the ACO, and the providers.”*
	Provide transportation support	*“Transportation…is one of the most difficult things that we deal with [day to day].”*

*Source*: Authors’ analysis of qualitative data from semistructured interviews of rural hospital executives.

*Note*: CMS is Centers for Medicare and Medicaid Services. AIM is ACO Investment Model. Discussion related to rural health clinics (RHCs) emerged in some interviews, since some hospitals own RHCs.

**TABLE 3:** Additional policy suggestions from rural hospital executives to (1) improve ACO effectiveness as well as (2) further attract and retain rural providers in ACO models, 2023–2024.

Categories	Suggestions	
**Modify ACO model components**	Remove the skilled nursing facility component for Medicare models	*“The skilled nursing care part [is] really very hard for rural communities because you don't have choices.”*
	Eliminate the high/low revenue designation in the MSSP	*“Get rid of that high/low revenue designation because…more ACOs would form if there was that advanced incentive payment.”*
**Adjust performance calculations**	Modify benchmarking, so it is not as hard to perform well in subsequent years	*“After…about year 3…with…the way the bar moves…you don't get shared savings back even though you're improving…diminishing returns, right?”*
	Continue emphasizing regional component in performance calculations	*“[Removing] national benchmarking—allowing…more influence on the regional side…should be advantageous to us.”*
**Improve patient communication and regulatory requirements**	Increase government‐led patient education	*“I think that the government should help educate the patients of what we are trying to do.…For example, we have chronic care management [patients] that sometimes…don't want to answer the phone call, and you understand that they might think it's just a scam.”*
	Reduce regulatory requirements that add cost without value	*“It's thousands of dollars to do mailings.…Things like that…don't [add] any benefit to our patients or the Medicare population.”*
**Hold patients more accountable**	Incorporate patient accountability	*“None of these plans really require the beneficiary to comply.…Accountability helps.”*
**Facilitate adequate care management staffing**	Enable critical access hospitals to have more patient advocates	*“People fall through the cracks…a lot of small critical access hospitals…might only have one patient advocate consultant.”*

*Source*: Authors’ analysis of qualitative data from semistructured interviews of rural hospital executives.

## 4 Discussion

Our results reveal multi‐faceted motivations drive rural hospitals to participate and remain in ACOs. Such motivations include (1) financial incentives—potential shared savings, upside‐only risk initially, and upfront funding for some providers, (2) resulting capacity of quality measures, data, and insights from other providers gained through ACO participation to drive improvements in population health and quality, (3) sense of urgency to get ahead of the shift toward value‐based reimbursement, (4) additional forms of resource gain, and (5) key outside factors (e.g., MACRA). Of these motivations, the first two were the most prevalent. These findings shed light on factors that may need to be at least partly preserved and potentially even enhanced for rural providers to remain in ACOs long‐term.

Our findings are consistent with prior research but also highlight lesser‐known motivations. Moreover, our research reveals cases where rural hospitals remain in ACOs despite the costs of care delivery changes or declines in hospital inpatient admissions (resulting from enhanced care management) exceeding financial gains from participation. This underscores the importance some respondents raised about the role for payers and providers to make patients more aware of the enhanced quality ACOs can provide. This awareness would help distinguish hospitals committed to achieving these gains, particularly for respondents investing in care delivery changes despite financial vulnerability.

This research highlights unique challenges rural providers face, hindering ACO participation and success, including smaller scale or populations, financial concerns, and limited capacity to bear downside risk. Such challenges can make it difficult to invest resources toward prevention and population health, even under ACO models. These observations are consistent with prior research findings that non‐metropolitan hospitals have significantly lower MSSP participation [8]. Additionally, respondents highlighted how reimbursement methods for certain rural providers (e.g., cost‐based payment for CAHs, capped payment for RHC visits) can affect participation in specific models. This suggests alternative ACO reimbursement designs could make ACO models more attractive to these providers. This raises questions such as whether to use an advanced payment method somewhat parallel to cost‐based payment, but a significant move away from cost‐based FFS payment.

ACO design affects rural providers’ decisions on the timing and selection of specific models. Many respondents started with the MSSP, because it was an early option and initially offered upside‐only risk. After gaining ACO experience, some respondents also joined, or switched to, commercial ACOs. For certain providers, such as CAHs who receive cost‐based reimbursement, some commercial ACO models were more financially attractive than Medicare models. One respondent specifically noted the magnitude of risk in the MSSP is significantly larger than their commercial contracts. This aligns with many rural hospitals’ concerns about MSSP risk driving their consideration to leave the MSSP or partner with ACO management organizations (e.g., Signify and Aledade).

Our findings highlight areas for policy refinement. The most prevalent recommendation was to reduce or eliminate the risk rural providers face when participating in ACO models. Beginning 2024, the MSSP offers a new, permanent payment option called Advance Investment Payments. This option encourages providers in rural and underserved areas to form ACOs by offering advance shared savings payments for investments in staffing, infrastructure, and care delivery improvements to enhance ACO performance [20, 21]. However, renewing or re‐entering ACOs are not eligible for this new payment option. Other prevalent recommendations included: improve access to timely data, modify quality measures to align with uniqueness of rural populations, increase reimbursement or incentives, and facilitate easier access to care (e.g., increase access to mid‐level providers, permit billing for a well visit and encounter the same day, eliminate chronic care management co‐pay, offer transportation assistance).

The design of ACO models must balance the benefits and drawbacks of one‐sided and downside risks—while ensuring the focus remains on enhancing quality and promoting population health without financially overburdening providers. One‐sided risk models encourage rural hospital participation by minimizing financial risks. However, concerns have been raised about their ability to drive long‐term care delivery changes due to limited financial stakes. Conversely, downside risk models place more financial pressure on hospitals with the goal of ensuring intended care delivery changes. However, our research, and others, show the financial burden and risk of these models lead many rural hospitals to leave ACOs or not enter them at all. Increasing rural provider participation will require refined incentives to ensure population health and quality improvements are financially feasible while minimizing financial threats. Without additional funding, continuing to invest efforts in large‐scale delivery of care changes to further improve population health and quality may not be feasible for financially vulnerable rural hospitals.

Together, these findings are vital to refine policy—to further increase the spread and retention of ACOs among rural providers. In turn, this can help achieve prominent goals for the future of health care.

## Funding

The research was supported by Arnold Ventures: Grant ID 22‐08114. The information, conclusions, and opinions expressed in this manuscript are those of the authors and no endorsement by Arnold Ventures is intended or should be inferred.

## Consent

The authors have nothing to report.

## Conflicts of Interest

We have no conflicts of interest
